# Ethyl Methane Sulfonate and Sodium Azide-Mediated Chemical and X-ray-Mediated Physical Mutagenesis Positively Regulate *Peroxidase 1* Gene Activity and Biosynthesis of Antineoplastic Vinblastine in *Catharanthus roseus*

**DOI:** 10.3390/plants11212885

**Published:** 2022-10-28

**Authors:** Vyoma Mistry, Pragya Tiwari, Paresh Patel, Gajendra Singh Vishwakarma, Geung-Joo Lee, Abhishek Sharma

**Affiliations:** 1 C. G. Bhakta Institute of Biotechnology, Maliba Campus, Uka Tarsadia University, Surat 394350, India; 2 Department of Biotechnology, Yeungnam University, Gyeongsan 38541, Korea; 3 Tarsadia Institute of Chemical Science, Uka Tarsadia University, Bardoli 394350, India; 4 Department of Biotechnology and Bioengineering, Institute of Advanced Research, Koba Institutional Area, Gandhinagar 392426, India; 5 Department of Horticulture, Chungnam National University, Daejeon 34134, Korea; 6 Department of Smart Agriculture Systems, Chungnam National University, Daejeon 34134, Korea

**Keywords:** *Catharanthus roseus*, chemical mutagenesis, ethyl methane sulfonate, monoterpenoid indole alkaloids, sodium azide, *PRX1*

## Abstract

*Catharanthus roseus* synthesizes bioactive therapeutic metabolites, known as monoterpenoid indole alkaloids (MIAs), including antineoplastic vinblastine and vincristine, which have high global demand, and antihypertensive ajmalicine, a serpentine. However, the in planta biosynthesis and accumulation of these phytopharmaceuticals are very low, attributed to their high cytotoxicity in the plant. Considering the low in planta concentration and over-harvesting of plant resources, biotechnological interventions have been undertaken to enhance the production of MIAs in plant systems. The present study was carried out to mutation through chemical and physical mutagenesis with sodium azide, ethyl methane sulfonate and X-rays, respectively, on *C. roseus* to determine their possible effects on the transcriptional modulation of MIA biosynthetic pathways in planta. The chemical mutagenesis resulted in delayed seed pod development in mutated *C. roseus* plants, with distinct leaf morphology and flower color. However, X-ray mutagenesis resulted in pollen-less sterile flowers. An HPLC analysis confirmed the higher catharanthine, vindoline and vinblastine content in sodium azide and X-ray mutants, and was further supported by higher *PRX1* transcript levels estimated through real-time PCR analysis. The transcription factors *WRKY1* and *ORCA2* were found negatively regulated along with major MIA pathway genes in chemical mutants and their M_1_ generation, but showed positive regulation in X-ray M_0_ mutants. The induced mutagenesis of *C. roseus* provides a prospective strategy to modulate plant transcriptomes and enhance the biosynthesis of pharmaceutically important antineoplastic vinblastine in the plant.

## 1. Introduction

**Figure 1:**
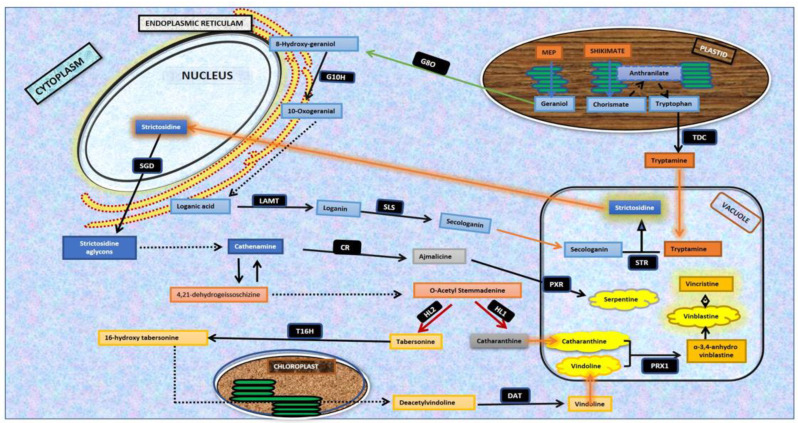
Schematic representation of monoterpenoid indole alkaloid (MIA) pathway in *C. roseus*. The following pathway genes/intermediates involved in MIA biosynthesis are shown as follows: G8O-, geraniol 8-oxidase; G10H-, geraniol 10-hydroxylase; LAMT-, loganic acid O-methyltransferase; *TDC*, tryptophan decarboxylase; SLS, secologanin synthase; STR, strictosidine synthase; *SGD*, strictosidine-β-D-glucosidase; *CR*, cathenamine reductase; *HL1*, *HL2*, hydrolase-type enzymes; *T16H*, tabersonine 16 hydroxylase; *DAT*, desacetoxyvindoline-4-O-acetyltransferase; *PRX1*, α-3′,4′ anhydrovinblastine synthase; *PRX*, peroxidase. Representative symbols: arrows; highlighted straight arrow: strictosidine transport from the vacuole to nucleus; straight arrow: direct enzymatic reaction; broken arrow: multistep enzymatic reactions with stable/unstable intermediates; double-ended arrows: isomerization steps; pathway genes are represented in black squares, intermediate molecules are represented in blue, orange and grey square boxes, and important metabolites are represented in clouds.

*Catharanthus roseus* (L.) G. Don. (Family Apocynaceae) is a highly important medicinal plant with immense medicinal value because of its potential to synthesize more than 130 known pharmaceutically active metabolites [1]. These key metabolites are designated as monoterpenoid indole alkaloids (MIAs) and comprise vinblastine and vincristine (antineoplastic), ajmalicine (antihypertensive), serpentine, catharanthine (reduction of blood sugar) and vindoline, which binds with catharanthine to produce 3,4-anhydroxyvinblastine, the precursor molecule of vinblastine. The discovery and commercialization of anticancer alkaloids, vincristine, and vinblastine have increased the demand for the enhanced production of MIA alkaloids and precursors, vindoline, and catharanthine [2], employing biotechnological interventions. The MIA biosynthetic pathway operates through a coordinated and regulated network of more than 30 enzymatic steps and 40 pathway intermediates. The dynamics and hierarchy of the MIA biosynthetic pathway assembly are evident by the requirement of four different cellular organizations (epidermis, laticifers, idioblast, and internal phloem-associated parenchyma) and five intracellular compartments (chloroplast, cytosol, nucleus, vacuole, and endoplasmic reticulum) to produce vinblastine and vincristine [1]. The complex and multi-step synthesis of MIA alkaloids includes tryptamine assembly with a secoiridoid, leading to strictosidine biosynthesis that works as a central intermediate, and is further chemically modified to produce diverse MIA analogs existing in nature [3] (Figure 1).

The multistep MIA biosynthetic pathway is strictly regulated at the transcriptional level through developmental, environmental, and spatial–temporal controls. Recently, the molecular mechanisms and transcriptional regulations of plant alkaloid biosynthesis have been extensively studied. Recent studies in elucidating the molecular networks in *C. roseus* have suggested a key network of transcription factors (TFs) that positively and negatively regulates the expression of major MIA pathway genes by either activating or repressing them [3]. The octadecanoid-responsive *Catharanthus* AP2/ERF-domain (ORCA) transcription factors (TFs), viz. ORCA 2, ORCA 3, ORCA 4, and ORCA 5, present as gene clusters on the same genome scaffold, positively regulating the leaf-specific alkaloids such as vindoline and catharanthine biosynthesis in *C. roseus* [4]. Studies deciphering the leaf, stem, root, and flower transcriptome analysis revealed the jasmonate-inducible nature of ORCA TFs, specifically in *C. roseus* hairy root cultures and cell suspension cultures [1]. Similarly, TFs WRKY 1 and WRKY 2 also demonstrated the positive regulation of root-specific MIA serpentine biosynthesis in *C. roseus* cell suspension cultures [5]. Thus, both ORCA and WRKY TFs demonstrate tissue-specific MIA regulation in *C. roseus*.

The emerging significance of MIAs in a wide range of pharmaceutical applications, including cardiovascular disorders, different kinds of cancers, and neurological diseases, has necessitated the detailed elucidation of metabolic pathways: MIA alkaloid biosynthesis and the transcriptional regulation in *C. roseus*. However, the strong cytotoxic nature of vinblastine and vincristine restricts *C. roseus* to accumulating these MIAs in higher concentrations, a key measure of the auto-defense of its physiological machinery, and has evolved complex spatial and temporal regulatory network for MIA pathway expression [6,7]. The presence of low amounts in plants/extracts and over-exploitation of the plant species has necessitated the need to explore alternative resources, and tremendous efforts have been made to gain insights into the biosynthesis of MIA alkaloids via genetic engineering approaches, bioreactor technologies, and cell cultures on a large scale for the enhanced production of high-value metabolites. For commercial production, semi-synthetic derivatives of vinblastine and vincristine are produced via monomeric precursors, catharanthine, and vindoline [8]. The present decade has witnessed extensive research into *C. roseus*, with substantial efforts being made to decode/delineate the missing links (steps) of the pathway architecture in terms of enzymes, intermediates, and key pathway genes [9], while metabolic modulations through hyper- or down-regulation of known MIA pathway genes are a primary focus of *C. roseus* biotechnological interventions [10]. Although great efforts are being made towards the improvement of the yield of plant alkaloids, mutagenesis for crop breeding/improvement via physical/chemical mutagens remains a less-explored area. Research efforts toward the development of efficient cell- and tissue-culture-based production platforms for MIAs under the influence of biotic and abiotic elicitations or precursor/intermediate feeding approaches are also becoming more precise and refined [11]. A less-explored yet interesting area in biotechnological interventions in *C. roseus* includes understanding the effect of physical and chemical mutagenesis in planta, and its impact on the genomics and metabolomics of MIA biosynthesis in *C. roseus*, defining the underlying concept for the present research work.

Mutagenesis is a method of choice in creating genetic variability in plants with less natural genotypic and phenotypic variations possessing a consistent genetic framework [12]. While mutagenesis may occur spontaneously in nature and is considered a driving force of evolution, induced mutagenesis can be achieved through laboratory procedures (by employing mutagens), leading to genetic alterations in biological genomes. The biotechnological interventions in *C. roseus* employing chemical mutagenesis have focussed on crop improvement and include key studies: ethyl methane sulphonate induced mutagenesis and enhanced antibacterial activity in the plant [13], the effect of gamma rays on plant growth and yield [14], and the mutation of chlorophyll in plant seeds [15], among others. In recent times, induced mutagenesis in plants has been employed to alter the qualitative and quantitative levels of metabolite biosynthesis by targeting the genetic architecture of plants, thereby regulating spatial and temporal biosynthesis of key phytomolecules and high-value metabolites. Physically and chemically induced mutagenesis defines an attractive approach to creating genetic variability and modulating metabolic pathways towards enhanced production of a targeted metabolite in planta [16]. Plant chassis via induced mutagenesis has become increasingly popular at present time as an effective approach to crop improvement [17], leading to improved plant traits and overall yields. In addition, induced mutagenesis in plants may be beneficial to modulating the biosynthesis of metabolites by targeting the genetic architecture of plants, controlling and regulating the spatial and temporal biosynthesis of important phytomolecules and metabolites. Studies in this direction have shown that gamma rays influence plant growth by affecting genetic, biochemical, and morphological changes in cells and tissues [18], while ethyl methyl sulphonate (EMS) has been reported to be an effective mutagen for the improvement of crop plants [19]. The benefits of induced mutagenesis depend on the effectiveness of mutagens and their efficiency, which is necessary for the targeted high frequency of desired mutations [20].

In the present study, we have induced chemical mutagenesis by employing the mutagens sodium azide (SA; NaN_3_) and ethyl methane sulfonate (EMS), and the physical mutagen X-ray, respectively. SA is a common bactericide, pesticide, and industrial nitrogen gas generator, and is known to induce high rates of mutation. SA, as an ionic compound, demonstrates its mutagenicity through an azide organic metabolite (analogous to L-azidoalanine) generated by the enzyme O-acetylserine sulfhydrylase [21,22]. This azide organic metabolite enters the cell nucleus and creates point mutation in genomic DNA through a predominant transition of G/C to A/T. Similarly, EMS is a strong alkylating agent that is known to modify nucleotides by alkylating guanine to O6-ethylguanine and leads to (mis)match pairing with thymine instead of cytosine, and thus altering G/C pairing to A/T pairing in subsequent DNA repair [23]. With a lower frequency, EMS can also cause G/C to C/G or G/C to T/A transversion and A/T to G/C transversion through 7-ethylguanine hydrolysis and (mis)matches of 3-ethyladenine, respectively [22,24]. The present study was carried out to explore the random chemical and physical mutagenesis by the mutagens SA, EMS, and X-rays to determine their effects on the transcriptional modulation of the monoterpenoid indole alkaloid pathway (via differential expression of key pathway genes and transcription factors in MIA biosynthesis) in *C. roseus* plants. Moreover, their physio-morphological and anatomical attributes with major MIA metabolites (viz., ajmalicine, catharanthine, vindoline, and vinblastine), pathway genes (*secologanin synthase* (*SLS*), *tryptophan decarboxylase* (*TDC*), *strictosidine synthases* (*STR*), *strictosidine β-D-glucosidase* (*SDG*), *tabersonine 16-hydroxylase* (*T16H*), *deacetylvindoline acetyltransferase* (*DAT*), *peroxidase* (*PRX1*)) and transcription factors, *WRKYGQK heptapeptide* at the *N-terminal end* (*WRKY1*) and *Octadecanoid-Responsive Catharanthus AP2-domain protein 2* (*ORCA2*) regulating MIA biosynthesis under a glass-house environment were determined. Considering the commercial success and huge global demands for the antineoplastic agents vinblastine and vincristine in cancer chemotherapies, chemically and physically induced mutagenesis of *C. roseus* defines an attractive approach to enhance in planta content of MIA alkaloids for pharmaceutical applications.

## 2. Results

### 2.1. SA, EMS, and X-ray Dose Optimization and Mutagenesis in M_0_ Generation

For mutagenesis studies, the seed germination rate of healthy seeds was estimated in the initial experiment. All 100 seeds showed germination, with almost 70% of seeds showing germination within the initial 4–5 days and the remaining 30% taking 7–8 days for the emergence of the radicle. After confirming the germination efficiency, the seeds were subjected to chemical mutagen SA and EMS treatment, initially for dose optimization and later for seed mutagenesis, to develop mutated plants. Under treatment of SA and EMS, seeds only mutated with a 0.1 mM to 0.5 mM concentration were able to manage 90–80% and 80–65% germination and survive more than 2 months, respectively. The optimization of sodium azide and ethyl methane sulphonate (chemical mutagens) dose concentration, and its effect on seed germination rate and plant survival, was determined.

The X-ray treatment was carried out in a completely randomized design with the availability of an X-ray energy range with the X-ray machine. The three different kV treatments used, viz., 55 kV, 102 kV, and 125 kV, indicate the low, moderate, and high X-ray energy intensity with different exposure time product values, i.e., 48, 96, and 128 mAs. The seedling and plant growth rate was found to be inversely proportional to the X-ray mutagen treatment. The seedlings mutated with 55 kV (low dose) showed a comparatively fast growth rate, followed by the 102 kV and 125 kV treatments, respectively. In terms of the plant growth, Rubisco and photosynthetic activity assessment of control plants suggested major plant growth and highest alkaloid content at the age of around 180 days; therefore, all the studies on the mutated plants were carried out at the age of 180 days.

### 2.2. Rubisco and Photosynthetic Activity Estimation

The mutated and control plants were maintained under greenhouse conditions, and the physio-morphological observations were recorded regularly. The mutated plants showed a much slower growth rate and biomass increase, which was inversely proportional to mutagen concentration. The plants mutated with a lower 0.1 mM mutagen concentration showed a growth rate and biomass increase compared to the control, whereas with an increase in mutagen concentration, plants showed a slower growth rate and biomass. The chlorophyll content was also recorded, which followed similar reduction trends as growth rate and biomass. In SA-treated plants, the highest total chlorophyll was recorded in SA 0.1, accumulated at 1.76 ± 0.29 mg/gm, whereas SA 0.5 accumulated the lowest at 1.23 ± 0.16 mg/gm. Among the EMS treatments, plants treated with EMS 0.1 accumulated higher (1.73 ± 0.24 mg/gm), and plants treated with EMS 0.5 accumulated lower (1.32 ± 0.19 mg/gm) total chlorophyll content.

However, all the treatments of SA and EMS accumulated lesser chlorophyll content compared to the control plant at 1.84 ± 0.19 mg/gm (Table 1). The mutated plants also recorded lower Rubisco activity compared to 4.40 ± 0.20 milliunits mL^−1^ mg protein^−1^ in the control plant. SA-mutated plants demonstrated Rubisco activity ranging from 3.58 ± 0.15 to 1.68 ± 0.08 milliunits mL^−1^ mg protein^−1^ in SA 0.1 and SA 0.5 treatments, and 3.05 ± 0.13 to 1.27 ± 0.12 milliunits mL^−1^ mg protein^−1^ in EMS 0.1 and EMS 0.5 treatments, respectively (Table 1).

X-ray-mutated plants also showed a similar trend of growth to SA- and EMS-mutated plants and showed comparatively slow growth and biomass increase, to control plants and were suggested to be inversely proportional to mutagen concentration. Plants mutated with 55 kV X-ray energy demonstrated almost equal growth to control; however, with the increase of X-ray dose to 102 kV and 125 kV, plants recorded slower growth rate and biomass (Table 1). The chlorophyll content followed similar reduction trends as growth rate and plant biomass. The highest total chlorophyll content was recorded as 1.08 ± 0.07 mg/g in 55 kV 89 mAs and 102 kV 48 mAs, while the 125 kV 48 mAs treatment recorded the lowest 0.54 ± 0.056 mg/g total chlorophyll content. However, all of the X-ray treatments accumulated lower chlorophyll content compared to the 1.09 ± 0.13 mg/g of control plants. The Rubisco activity of all mutated plants was also recorded as lower compared to the 2.86 ± 0.28 milliunits mL^−1^ mg protein^−1^ in control. Furthermore, Rubisco activity also demonstrated similar trends as chlorophyll content, and gradually reduced with the increase in the X-ray doses. In mutated plants, the highest and lowest Rubisco activity was observed from 2.84 ± 0.28 milliunits mL^−1^ mg protein^−1^ to 1.63 ± 0.14 milliunits mL^−1^ mg protein^−1^ in the 102 kV 48 mAs and 125 kV 98 mAs treatments, respectively (Table 1).

### 2.3. Histological and Morphological Analysis

**Figure 2:**
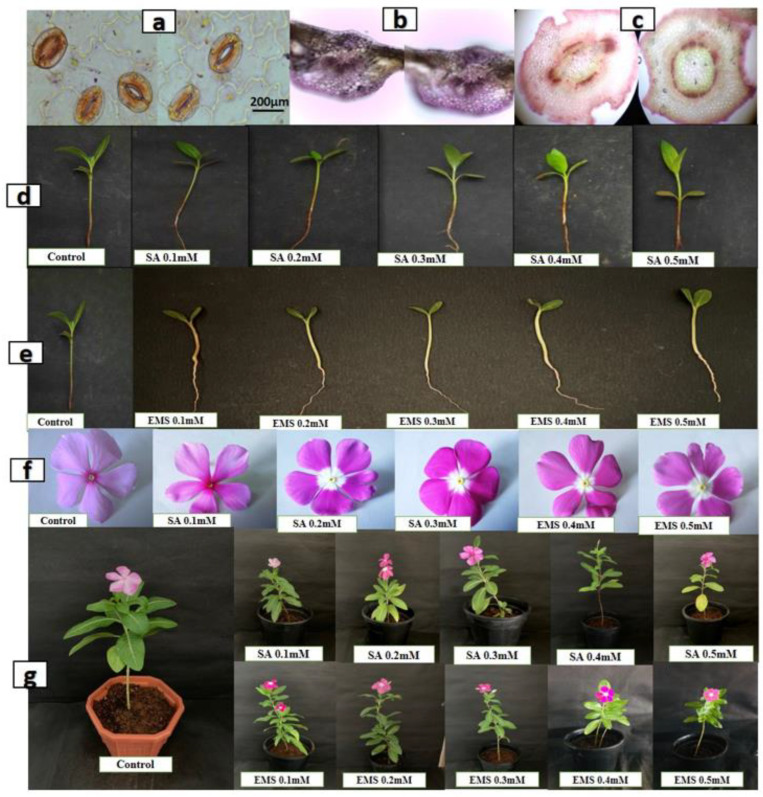
Microscopic and morphological observations of SA- and EMS-mutated and control *C. roseus* plants. The figures are represented as follows: (**a**) The thick-walled epidermis cell was irregularly organized and the stomatal assemblies with both anomocytic and anisocytic stomata were seen embedded in amoeboid epidermal cells. (**b**) The compact palisade layer was found beneath the upper epidermis with 2–4 layers of spongy mesophyll cells, and the vascular bundle found was conjoint, collateral and closed, containing xylem towards the lower and phloem towards the upper epidermis in both mutated and control plants. (**c**) The vascular bundle was encircled with endodermis in both mutated and control plants, with phloem having thin-walled fiber cells. The pith was comprised of isodiametrical parenchyma and idioblast cells, including a major portion of the stem (**d**,**e**,**g**), and the chemical mutagenesis by both SA and EMS affected the seed germination, plant growth and morphology. (**f**) The chemical mutagenesis also affected the flower pigmentation. The control shows a pink flower with a dark pink central ring, whereas the mutagenesis resulted in different flower petal structure, shape and size, with the white color at the bottom of the petals attached to the central ring having random pink and yellow colors.

Histological analysis of the leaf epidermis, transverse section (TS) of leaf midrib, and stem of both mutated and control plants was performed. The microscopic examination of the leaf epidermis revealed its single-layered structure. The thick-walled epidermis cell was irregularly organized and the stomatal assemblies with both anomocytic and anisocytic stomata were seen embedded in amoeboid epidermal cells (Figure 2a). The anatomical observation of leaf TS showed the general arrangement of leaf cells and tissues with no significant difference. The compact palisade layer was found beneath the upper epidermis with 2–4 layers of spongy mesophyll cells, and the vascular bundle found was conjoint, collateral, and closed, containing xylem towards the lower and phloem towards the upper epidermis in both mutated and control plants (Figure 2b). TS of the stem showed a major difference in the distribution of idioblast cells, which was regular in control but with irregular occurrence in mutated plants.

**Figure 3:**
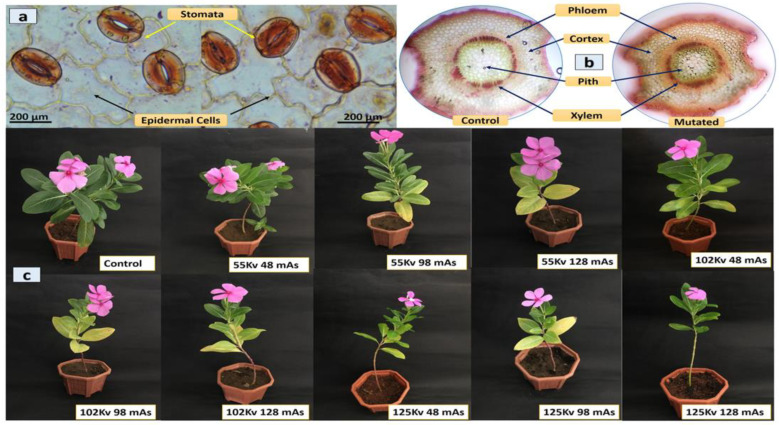
Microscopic and morphological observations of mutated and control *C. roseus* plants. The figures are represented as follows: (**a**) The thick-walled epidermis cell was irregularly organized and the stomatal assemblies with both anomocytic and anisocytic stomata were seen embedded in amoeboid epidermal cells. (**b**) The vascular bundle was encircled with endodermis in both mutated and control plants, with phloem having thin-walled fiber cells. The pith was comprised of isodiametrical parenchyma and idioblast cells, including a major portion of the stem. (**c**) The physical mutagenesis by X-rays affected the plant growth and morphology.

The vascular bundle was encircled with endodermis in both mutated and control plants, with phloem having thin-walled fiber cells. The pith was comprised of isodiametric parenchyma and idioblast cells, including a major portion of the stem (Figure 2c). The chemical mutagenesis by both SA and EMS affected the seed germination, plant growth, and morphology. The increase in the doses of SA and EMS reduced plant growth with minor changes in leaf morphology (Figure 2d,e,g). The leaves of mutated plants were found to be less broad and elongated compared to the control. Additionally, chemical mutagenesis affected flower pigmentation. The control showed a pink flower with a dark pink central ring, whereas the mutagenesis resulted in different flower petal structure, shape, and size, with the white color at the bottom of the petals attached to the central ring having random pink and yellow colors (Figure 2f). The transverse section of the stem did not show a major difference in cellular arrangement in X-ray-mutated and control plants. The vascular bundle was found encircled with endodermis, and phloem with thin-walled fiber cells. The pith was comprised of iso-diametrical parenchyma and idioblast cells, including a major portion of the stem. The leaves of mutated plants were found to be slightly broad, elongated, and pointed compared to the control. No major difference was observed in the flower pigmentation (Figure 3). However, the effect of mutagenesis was observed in the seed production post-flowering. *C. roseus* is a self-pollinating plant; however, the seed pods were not developed in all mutated plants for more than almost 12 months. The manual pollination of flowers was also attempted using the needle rolling approach in flowers to assist pollination. However, at the age of 14 months, SA- and EMS-mutated plants developed seed pods; however, X-ray-mutated plants failed to produce seed pods and died at the age of 18 months.

### 2.4. HPLC Quantification of MIAs Alkaloids

The study was performed for comparative analysis of SA- and EMS-mutated M_0_, M_1,_ and control plants via HPLC analysis of major MIAs, viz., ajmalicine, catharanthine, vindoline, and vinblastine as summarized. In the MIA pathway, the cathenamine produced from strictosidine aglycones diverts the pathway flux in two directions, first towards ajmalicine, which produces serpentine, and second towards 4,21-dehydrogeissoschizine, which, further by a series of several enzymatic steps, produces o-acetyl stemmadenine, that again diverts pathway flux in two directions. The first diversion from o-acetyl stemmadenine produces tabersonine that leads to the biosynthesis of vindoline, and the second diversion produces catharanthine, the last monomeric MIA and precursor of vinblastine. The HPLC analysis of M_0_ SA-mutated plants showed lower ajmalicine accumulation, where only SA 0.5 showed slightly enhanced ajmalicine accumulation of 0.031 ± 0.003% dry weight in SA 0.5-treated plants compared to 0.029 ± 0.003% dry weight in control.

In EMS-treated M_0_ plants, EMS 0.1-mutated plants accumulated 2-fold higher 0.031 ± 0.003% dry weight ajmalicine, followed by 0.049 ± 0.003% dry weight and 0.035 ± 0.003% dry weight in EMS 0.5- and EMS 0.2-mutated plants, respectively. Both SA 0.4- and EMS 0.4-mutated plants accumulated less ajmalicine compared to the control.

**Figure 4:**
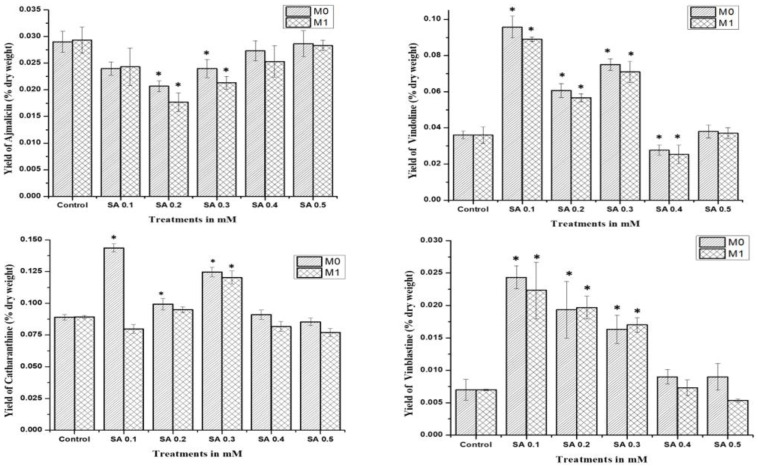
HPLC analysis of the key MIAs ajmalicine, catharanthine, vindoline, and vinblastine in SA-mutated M_0_–M_1_ and control *C. roseus* plants. * represents a significant difference from the respective control at *p* < 0.05 after applying post hoc Dunnett’s test (*n* = 3).

The catharanthine accumulation was found to enhance in SA- and EMS-mutated M_0_ plants. SA 0.1 accumulated 0.096 ± 0.008% dry weight catharanthine, with an almost 3-fold increase compared to 0.036 ± 0.004% dry weight in control, followed by an almost 2-fold increase of 0.075 ± 0.007% dry weight in SA 0.3, and 0.061 ± 0.007% dry weight in SA 0.2, whereas EMS 0.5 showed the highest 3-fold increase in catharanthine accumulation, followed by a more than 2-fold increase of 0.086 ± 0.008% dry weight in EMS 0.1, and a 1.5-fold increase of 0.054 ± 0.007% dry weight in EMS 0.2 and 0.059 ± 0.007% dry weight in EMS 0.3, whereas EMS 0.4 accumulated far less (0.008 ± 0.001%) dry weight catharanthine, and SA 0.4 and EMS 0.4 accumulated less catharanthine compared to the control (Figure 4). Vindoline accumulation was also observed to be higher in M_0_-mutated plants, compared to the control. Among the SA treatments, SA 0.1 and SA 0.3 showed the highest vindoline accumulation, 0.144 ± 0.012% dry weight, and 0.125 ± 0.011% dry weight, respectively, compared to 0.089 ± 0.09% dry weight in control, whereas in EMS treatments EMS 0.5 accumulated the highest vindoline 0.148 ± 0.012% dry weight, followed by EMS 0.1 with 0.131 ± 0.011% dry weight. However, both SA 0.4 and EMS 0.4 accumulated less vindoline compared to the control. Vinblastine content was observed in all M_0_ SA- and EMS-mutated plants, except EMS 0.4-treated plants.

The highest content of more than 3-fold enhanced vinblastine 0.24 ± 0.003% dry weight was observed in SA 0.1, followed by a more than 2-fold increase of 0.19 ± 0.003% dry weight and 0.16 ± 0.003% dry weight in SA 0.2 and SA 0.3, respectively. In addition, EMS 0.5 also accumulated 3-fold enhanced vinblastine 0.21 ± 0.003% dry weight, followed by a more than 2-fold increase of 0.17 ± 0.003% dry weight and 0.16 ± 0.003% dry weight in EMS 0.3 and EMS 0.1, respectively.

**Figure 5:**
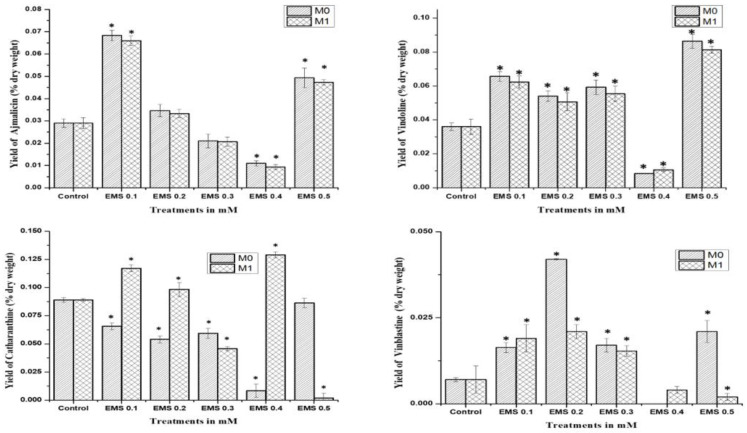
HPLC analysis of the key MIAs ajmalicine, catharanthine, vindoline, and vinblastine in EMS-mutated M_0_–M_1_ and control *C. roseus* plants. * represents a significant difference from the respective control at *p* < 0.05 after applying post hoc Dunnett’s test (*n* = 3).

The mutagenic effect of SA, EMS, and X-rays drastically affected the seed production in the mutated M_0_ generation, where only SA and EMS mutants provided seeds for the M_1_ generation after almost 14 months of age. HPLC analysis of SA and EMS mutants of the M_1_ generation followed the same pattern of M_0_ mother plants for ajmalicine, catharanthine, vindoline, and vinblastine biosynthesis (Figure 5). All SA M_1_ mutants accumulated marginally fewer amounts of ajmalicine compared to the control, whereas the highest vindoline and catharanthine content were obtained in SA 0.1 followed by the SA 0.3 mutant line. Vinblastine content was found highest in the SA 0.1 mutant line, followed by SA 0.2 and so on. Similarly, M_1_ EMS mutants demonstrated the same pattern as EMS M_0_ mutants, where the highest ajmalicine, catharanthine, vindoline, and vinblastine accumulated in EMS 0.1, EMS 0.5, EMS 0.4, and EMS 0.2 mutants, respectively. This specific pattern of MIA accumulation in M_0_ and M_1_ suggests the impact of mutagenesis on physio-genetic architecture.

**Figure 6:**
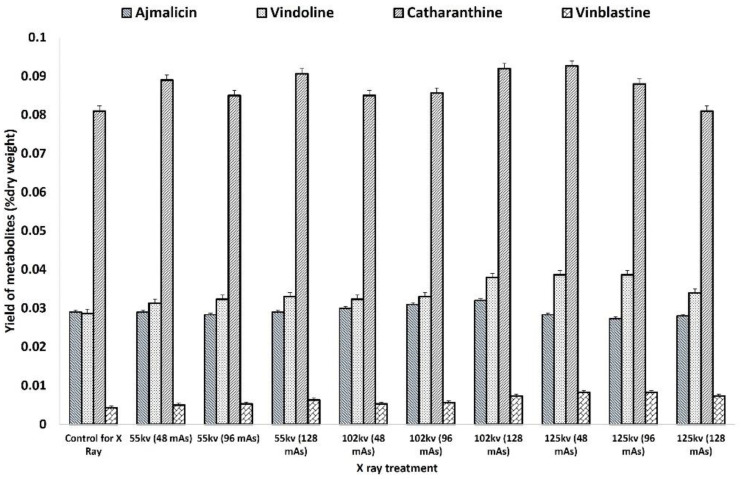
HPLC analysis of the key MIAs ajmalicine, catharanthine, vindoline, and vinblastine in X-ray-mutated M_0_ and control *C. roseus* plants. No significant difference was observed from the respective control at *p* < 0.05 after applying post hoc Dunnett’s test (*n* = 3).

The HPLC analysis of X-ray-mutated and control plants recorded the comparatively higher accumulation of antihypertensive ajmalicine, antineoplastic dimeric MIA vinblastine, and its monomeric precursors vindoline and catharanthine. The accumulation of ajmalicine was found to be equal and slightly higher in all treatments of 55 kV and 102 kV except for higher doses of 125 kV (Figure 6). The highest ajmalicine was recorded at 0.032 mg % dry wt. compared to 0.028 mg % dry wt. in control, whereas the accumulation of vindoline, catharanthine, and vinblastine was found to be consistently higher in mutated plants compared to the control. The highest catharanthine content of 0.039 mg % dry wt. and 0.038 mg % dry wt. was observed in 125 kV- and 102 Kv-mutated plants, respectively, compared to 0.029 mg % dry wt. in control, whereas the highest vindoline content of 0.092 mg % dry wt. was recorded in 102 kV- and 125 kV-mutated plants. Similarly, the highest accumulation of vinblastine 0.008 mg % dry wt., 2-fold more than the control 0.004 mg % dry wt., was recorded in the 102 kV- and 125 kV-mutated plants.

### 2.5. Real-Time PCR Quantification

**Figure 7:**
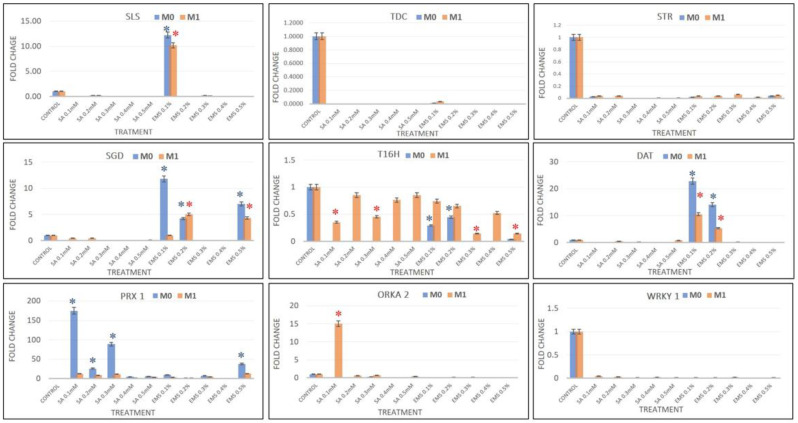
Real-time PCR quantification of the key MIA pathway genes in *C. roseus* in different concentrations of SA- and EMS-treated and control plants. The pathway genes are represented as: *SLS*, secologanin synthase; *TDC*, tryptophan decarboxylase; *STR*, strictosidine synthase; *SGD*, strictosidine b-D-glucosidase; *T16H*, tabersonine 16-hydroxylase; *DAT*, deacetylvindoline acetyltransferase; *PRX1* peroxidase, and transcription factors- *ORCA2*, and *WRKY1*, key regulators of the MIA pathway, were determined using Livak’s 2^−ΔΔCt^ method (along with controls). The Ct value (threshold cycle) for each gene was normalized against the Ct from *C. roseus* RSP9 gene. (Primer list provided in Supplementary Materials). * represents that a significant difference was observed from the respective control at *p* < 0.05 after applying post hoc Dunnett’s test (*n* = 3).

Real-time PCR analysis was performed to chase the effect in SA and EMS M_0_–M_1_ mutants in terms of relative transcript expression of eight candidate MIA pathway genes, viz., *SLS*, *TDC*, *STR*, *SGD*, *T16H*, *DAT*, *PRX1*, and two transcription factors, *ORCA2* and *WRKY1*, and compared with the results of HPLC analysis. The real-time PCR data analysis revealed the downregulation of *SLS* in all SA- and EMS-mutated M_0_ and M_1_ plants, except EMS 0.2, which showed more than 10-fold enhanced *SLS* expression, compared to the control. Additionally, the major genes *TDC* and *STR* were downregulated in all treatments, suggesting a low pathway flux pressure towards MIA central intermediates and strictosidine in both M_0_ and M_1_ mutant lines. Strictosidine is the central intermediate of the entire MIA pathway that leads to the production of all different types of MIAs, including vinblastine and vincristine. Strictosidine is converted to strictosidine aglycone by the activity of *SGD*. The expression levels of *SGD* were found downregulated in all SA treatments; however, EMS 0.1, EMS 0.5, and EMS 0.2 showed enhanced *SGD* levels in both M_0_ and M_1_ mutants. The expression of *T16H* and *DAT* genes were found to be downregulated, except for EMS 0.1 and EMS 0.2, which showed more than 24-fold and 14-fold *DAT* expression in M_0_ mutants, and were more than 4-fold enhanced in M_1_ mutants. However, the levels of *PRX1* gene expression were observed in all treatments except the EMS 0.4 M_0_ mutant, but all M_1_ mutants demonstrated enhanced *PRX1* transcripts compared to the control but lower compared to their M_0_ parent (Figure 7). The condensation step coupling vindoline and catharanthine towards vinblastine was facilitated by *PRX1*. The SA 0.1 dramatically showed the highest 174-fold enhanced *PRX1* expression, followed by more than 88-fold and 25-fold in SA 0.3 and SA 0.2, respectively. EMS 0.5 reported 37-fold enhanced *PRX1* overexpression, followed by more than 9-fold and 7-fold in the EMA 0.1 and 0.3 treatments. Therefore, the enhanced accumulation of vinblastine in all the mutated plants was found in direct proportion to the expression levels of *PRX1*, except in EMS 0.4, where both *PRX1* expression and vinblastine were not accumulated. Along with the expression levels of MIAs genes, the expression of transcription factors *ORKA2* and *WRKY1* was also studied, which was found negatively regulated in correlation to vinblastine biosynthesis.

**Figure 8:**
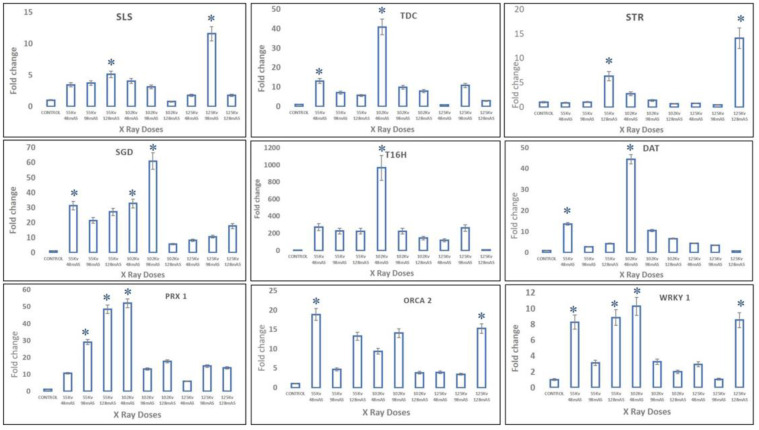
Real-time PCR quantification of the key MIA pathway genes in *C. roseus* in different X-ray-treated and control plants. The pathway genes are represented as: SLS, secologanin synthase; TDC, tryptophan decarboxylase; STR, strictosidine synthase; SGD, strictosidine b-D-glucosidase; T16H, tabersonine 16-hydroxylase; DAT, deacetylvindoline acetyltransferase; PRX1 peroxidase, and transcription factors- ORCA2, and WRKY1, key regulators of the MIA pathway, were determined using Livak’s 2^−ΔΔCt^ method (along with controls). The Ct value (threshold cycle) for each gene was normalized against the Ct from *C. roseus* RSP9 gene (primer list provided in Supplementary Materials). * represents that a significant difference was observed from the respective control at *p* < 0.05 after applying post hoc Dunnett’s test (*n* = 3).

In X-ray-mutated plants, transcript levels of upper MIA pathway genes, viz., *SLS*, *TDC*, and *STR*, were observed at maximum, with almost 11-fold, 40-fold, and 14-fold enhancement in 125 kV 98 mAs-, 102 kV 48 mAs-, and 125 kV 128 mAs-mutated plants, whereas the middle MIA pathway genes, viz., *SGD*, *T16H*, and *DAT*, recorded the highest expression levels in 102 kV 98 mAs- and 102 kV 48 mAs-mutated plants, respectively. Moreover, slightly different from trends of upper- and mid-MIA pathway genes, the highest expression levels of more than 45-fold of the final MIA pathway gene *PRX1* were recorded in 102 kV 48 mAs- and 55 kV 128 mAs-mutated plants. The transcription factors *WRKY1* and *ORCA2* recorded more than 18-fold and 10-fold enhanced transcript levels in 102 kV 98 mAs- and 55 kV 48 mAs-mutated plants, respectively (Figure 8).

## 3. Discussion

Recent initiatives towards enhancing the in planta concentrations of MIAs have focussed on raising in vitro *C. roseus* cultures [25], bioreactor-based systems [26], genetic engineering of *C. roseus* cultures to increase MIA production [27], and metabolic engineering for MIA production [28], among other interventions [29]. Moreover, the molecular elucidation for identification of the missing steps (enzymes, genes, and intermediates) being successfully deciphered [9], and the metabolic alterations by up/downregulation of genes in the MIA pathway, form the key areas in biotechnological interventions at the present time, while the metabolic engineering approaches in *C. roseus* have focussed on improving metabolic flux through precursor feeding, heterologous expression of genes, and use of elicitors, respectively.

Physically and chemically induced mutagenesis defines an attractive approach to creating genetic variability and modulating metabolic pathways towards enhanced production of a targeted metabolite in planta [16]. Plant chassis via induced mutagenesis has become increasingly popular in the present time as an effective approach to crop improvement [17], leading to improved plant traits and overall yields. In addition, induced mutagenesis in plants may be beneficial to modulating the biosynthesis of metabolites by targeting the genetic architecture of plants, controlling and regulating the spatial and temporal biosynthesis of important phytomolecules and metabolites. Studies in this area have shown that gamma rays influence plant growth by affecting genetic, biochemical, and morphological changes in cells and tissues [18], while ethyl methyl sulphonate (EMS) has been reported to be an effective mutagen for the improvement of crop plants [19]. The benefits of induced mutagenesis depend on the effectiveness of mutagens and their efficiency, which is necessary for the targeted high frequency of desired mutations [20].

The present work discusses the effect of chemical and physical mutagens, viz., SA-, EMS-, and X-ray-induced mutagenesis in *C. roseus* cv. Dhawal and its effect on the transcriptional modulation of the monoterpenoid indole alkaloid pathway (via differential expression of key pathway genes and transcription factors in MIA biosynthesis). The seeds of *C. roseus* cv. Dhawal were tested for seed germination assessment to select the best germinating seeds for the mutagen treatments. The entire seed population recorded germination within the range of 7–8 days, showing the good quality of the plant seeds. Mutagen dose optimization for mutagenesis suggested the use of 0.1 mM to 0.5 mM and 0.1% to 0.5% concentration of SA and EMS, respectively. X-ray treatment was carried out using a SIEMENS MULTIX X-ray system coupled with a Multiphos-450 X-ray generator, which is frequently used in medical diagnostic practice. X-ray doses given to the seeds were the minimum exposure doses possible from the machine after consulting with the radiologist. The seedlings mutated with 55 kV showed a comparatively fast growth rate, followed by 102 kV and 125 kV, respectively. Therefore, delay in the seed germination rate, along with plant growth rate, was found inversely proportional to the mutagen treatment. Seed germination and plant growth rate in mutagen-treated seeds defined the major parameters to evaluate the biological influence of mutagens over plant genetics controlling physio-morphological processes and metabolism [30,31,32]. Seeds treated with lower mutagen concentrations showed more and faster germination as well as growth, compared to seeds treated with higher mutagen concentrations. The possible reason behind this physio-morphological process remains the hindrance in the genetic and cytological process associated with enzymes, facilitating the seed germination and metabolism [2] and mitotic disruption through inter-chromosomal chromatin fibers, resulting in the sub-chromatid joining of chromosomes known as C-metaphase configuration [33].

Furthermore, the total chlorophyll content, along with chlorophyll a and chlorophyll b, was found to be reduced in the mutant plants and followed similar trends to seed germination and plant growth that can be attributed to the physio-morphological stress on mutated plant machinery. In jasmonic acid elicitation studies on *C. roseus*, Wei et al. [34] inferred that MIA pathway genes expression was directly proportional to the associated cytochrome P450 enzyme (*CPR*) expression. Additionally, most MIA pathway genes are associated with mitochondrial and chloroplast membranes [1,10]. In addition, the cytochrome P450 monooxygenase system is associated with the photosynthetic membrane and mitochondrial membrane, regulating the photosynthesis and electron transport chain through the light absorption capacity of chlorophyll pigments and resulting light-inducible nature of cytochrome enzymes. Along similar lines, Christ et al. [35] further demonstrated that the cytochrome P450 monooxygenase system associated with the formation of non-fluorescent chlorophyll catabolites (NCCs, a chlorophyll breakdown product in *Arabidopsis thaliana*) in dying cells or cells under stress conditions. Therefore, the downregulation of TFs and major MIA pathway genes in the present study can be interpreted as the formation of NCCs, and might be the reason behind the lower chlorophyll content in mutated plants. However, presently it remains a hypothesis and needs to be studied more to bridge the correlation between chlorophyll content and MIA pathway gene expression. Similar to chlorophyll content, the mutated plants also recorded lower Rubisco activity. Rubisco not only plays an important role as a photosynthetic enzyme, but it is also considered to be a nitrogen-storing protein [36]. Being a nitrogen-storing protein, upon degradation, Rubisco liberates nitrogen that is diverted to the developing plant organs [37]. Therefore, the functional relation of chlorophyll content, photosynthetic activity, and Rubisco content can be correlated.

The chemical mutagenesis by both SA and EMS affected the leaf and flower morphology with the increase in chemical doses. The change in the flower colors suggested a possible mutation in the genes associated with the flavonoid biosynthetic pathway [38]. The major problem associated with the SA EMS mutagenesis study restricted the screening of the mutants in M_0_ and M_1_ generations only. The effect of the mutagenesis was on the seed production capabilities of the mutated plants. *C. roseus* is a self-pollinated plant; however, the flowers developed in SA- and EMS-mutated plants failed to develop seed pods for more than almost 12 months and using the manual approach through manual needle rolling to assist self-pollination, whereas X-ray-mutated plants failed to develop seed pods and died at the age of around 18 months. Therefore, at 12 months of age, SA M_0_ mutants provided seeds for the M_1_ generation and thus molecular screening of only the SA M_1_ generation was carried out. M_1_ generation of SA-mutated plants also remained non-seed-productive until the age of 12 months; therefore, the genetic screening of mutants to study the heredity of mutational changes became the most problematic part of the study. The morphological examination of the flower revealed the pollen-less male-sterile mutant nature of the flower that restricted the pollination in the flower, hence no seed formation occurred. This interpretation was consistent with the studies of Kulkarni and Baskaran [39], who obtained similar results in EMS-induced mutagenesis in *C. roseus*, where instead of normal ‘thrum’ flowers (stigma position below the anthers), two different types of mutant flowers, one a pollen-less male-sterile mutant and the other a ‘pin’ flower with long-styled stigma above the anthers, were obtained [39].

The MIA biosynthesis traced via HPLC analysis of the control and mutated plants demonstrated the comparatively higher accumulation of antineoplastic dimeric MIA vinblastine and its monomeric precursors, catharanthine and vindoline. The accumulation of ajmalicine was found to be slightly lower in SA treatments but comparatively higher in EMS treatments. However, their correlation with MIA pathway gene expression analysis was in contrast to SA- and EMS-mutated and X-ray-mutated plants. Both M_0_ and M_1_ generations of SA- and EMS-mutated plants followed the same trends in terms of MIA biosynthesis and gene expression analysis. Their real-time PCR analysis revealed the downregulation of early- and mid-MIA biosynthetic pathway genes viz. *SLS, TDC, STR, SGD, T16H* and *DAT* in the most mutated plants, along with total downregulation of transcription factors *WRKY1* and *ORCA2*, whereas X-ray-mutated plants recorded MIA-enhanced biosynthesis along with enhanced transcript levels of *SLS, TDC, STR, SGD, T16H, DAT*, and *PRX1*, and TFs *WRKY1* and *ORCA.* However, the expression of the PRX1 gene facilitating the coupling of vindoline and catharanthine to form vinblastine was found to be enhanced in all SA-, EMS-, and X-ray-mutated plants. *SLS* is one of the important pathway genes leading to the formation of secologanin and its expression can lead to enhanced secologanin formation, required for the coupling with tryptamine to produce the central MIA intermediate compound strictosidine; additionally, the expression levels of *TDC* and *STR* were also found to be downregulated. This condition describes the low pathway flux ‘push’ towards strictosidine biosynthesis, as discussed earlier [1]. The expression levels of SGD were found to be downregulated in all SA and EMS except EMS 0.1, 0.2, and 0.5, suggesting a re-direction of the available pathway flux towards the cathenamine, which again diverts the MIA pathway flux towards ajmalicine and 4,21-dehydrogeissoschizine. The 4,21-dehydrogeissoschizine is further processed by a series of enzymatic steps and converted to o-acetyl stemmadenine, which diverts the pathway flux first towards tabersonine, leading to biosynthesis of vindoline, and secondly towards catharanthine. The comparative enhanced production of ajmalicine and catharanthine in EMS treatments are correlated to enhanced levels of *SGD* overexpression; however, enhanced ajmalicine and catharanthine might be present due to the natural physiological state of the plant, allowing their accumulation against pathway gene downregulation. Under the influence of elicitation and gene overexpression studies in cell suspension, transient (callus and leaves) and whole-plant transgenics of *C. roseus* expression of *SGD* demonstrated the pathway flux ‘push’ towards downstream MIAs [27,40,41].

In X-ray-mutated plants, correlation of the HPLC results with real-time PCR analysis revealed the upregulation of early-, mid- and lower-MIA pathway genes, along with TFs, *WRKY1*, and *ORCA2* in mutated plants. *SLS* and the *TDC* are one of the most important MIA early-pathway genes, leading to the formation of secologanin and tryptamine, respectively. Their overexpression leads to enhanced secologanin and tryptamine formation, followed by their coupling to produce the central MIA intermediate strictosidine [7,27]. In the present study, the enhanced expression levels of *SLS, TDC*, and *STR* were recorded, which allows the concentration of Seco(iridoid) and MEP pathway flux towards the central MIA intermediate strictosidine. This condition described the pathway flux ‘push’ towards strictosidine biosynthesis, as previously discussed [1]. Enhanced expression levels of *SGD* also suggested the re-direction of the available pathway flux towards the cathanamine. Cathanamine diverts the MIA pathway flux towards ajmalicine and 4,21-dehydrogeissoschizine. The 4,21-dehydrogeissoschizine tends to be processed by a series of enzymatic steps and converted to O-acetyl stemmadenine that diverts the pathway flux first towards tabersonine, leading the to biosynthesis of vindoline, and secondly towards catharanthine. The comparative enhanced production of ajmalicine, vindoline, and catharanthine in mutated plants correlated with enhanced *SGD* and *T16H* transcript levels. In several studies carried out under the influential conditions of elicitation and gene overexpression in cell suspension, and transient (callus and leaves) and whole plant transgenics of *C. roseus*, over-expression of *SGD* and *T16H* demonstrated the pathway flux ‘push’ towards downstream MIAs [27,40,41].

**Figure 9:**
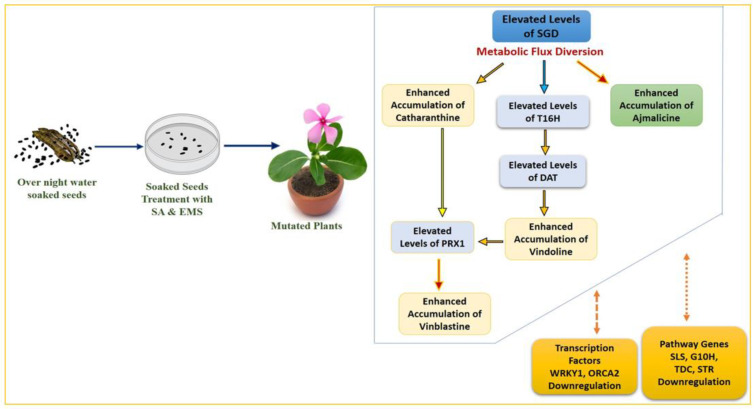
Mutagenesis’ possible correlation with MIA content and gene expression in SA- and EMS-mutated plants.

**Figure 10:**
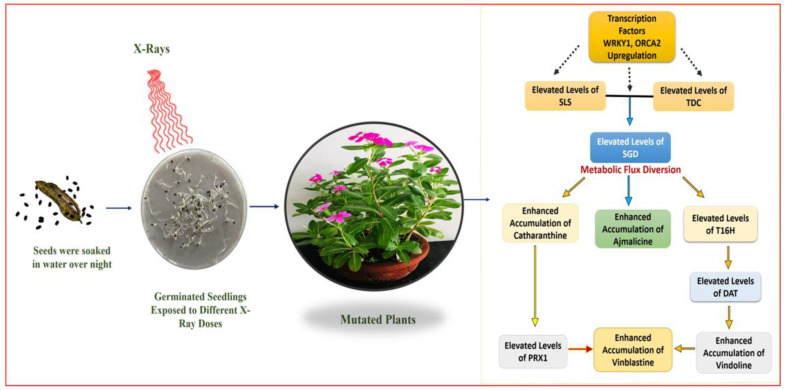
Mutagenesis’ possible correlation with MIA content and gene expression in X-ray-mutated plants.

Despite the differentiated correlation between MIA biosynthesis and expression of MIA pathway genes and related TFs in SA, EMS, and X-rays, the most important observation was the overexpression of the *PRX1* gene in all mutated plants. The presence of abundant vindoline and catharanthine levels along with enhanced *PRX1* levels facilitated the formation of vinblastine in all the mutated plants, except the EMS 0.4-mutated plants, where the reduced accumulation of vindoline and catharanthine was observed, and the plant failed to form vinblastine due to the absence of *PRX1* activity. This correlation of vinblastine formation with vindoline, catharanthine, and *PRX1* levels was established and discussed in detail in recent metabolic engineering studies [27,41,42]. Wang et al. [42] and Sharma et al. [27,41] recorded similar trends of *PRX1* activity, where high *PRX1* levels facilitated the enhanced vindoline and catharanthine conversion into vinblastine at the elicited and genetically engineered transient levels (in callus and leaves) as well as the whole plant transgenic level in *C. roseus*. Thus, the role of enhanced *PRX1* levels to induce the pathway flux ‘pull’ towards downstream of the MIA pathway to produce an adequate amount of antineoplastic dimeric MIA vinblastine in the presence of enhanced levels of its monomeric precursors, vindoline and catharanthine, can be conclusively inferred. In the present study, downregulation of *ORCA1* and *WRKY2* TFs was seen in SA- and EMS-mutated plants whereas it was found to be upregulated in X-ray-mutated plants. Several studies on elicitation and homo/heterologous MIA gene expression in *C. roseus* attempted to explore the role of transcription factors and MIA biosynthetic pathway genes with respect to MIA metabolites, specifically with vindoline, catharanthine, and vinblastine. Under many of these studies, the transcription factors ORCA and WRKY were reported to be the positive regulators of MIA pathway genes, such as *SLS, TDC, STR, D4H, CPR*, etc. [5,43,44,45,46,47]; however, their correlation in terms of *PRX1* has not yet been studied. However, the majority of these studies have been carried out with cell suspension, callus, and hairy root cultures, which do not provide the required complex cellular and subcellular organization and spatial–temporal regulation required for the completion of the entire MIAs biosynthetic pathway, hence, do not provide the in-depth correlation of MIA gene expression and metabolite biosynthesis [1,42,48,49]. Therefore, it can be concluded that the structural and genetic regulation limitations of previous studies restrict their comparative results’ correlation with the results obtained from induced mutagenesis in the present study (Figure 9 and Figure 10).

## 4. Materials and Methods

### 4.1. Plant Material and Seed Germination Assessment

The seeds of *C. roseus* cv. Dhawal (National Gene Bank Accession Number, CIMAP-0859; Council for Scientific and Industrial Research—Central Institute of Medicinal and Aromatic Plants, Lucknow, India; www.cimap.res.in) were used for the present study. The collected 100 seeds were cleaned 4–5 times with distilled water to remove germination inhibitors followed by surface sterilization with 0.1% HgCl_2_ for 60 s, followed by multiple washing with sterilized distilled water. Seeds were allowed to germinate in 90 mm sterile glass Petri-plates lined with Whatman No.1 filter paper moistened with sterile tap water to ensure adequate moisture for the seeds in a seed germinator at 34 ± 1 °C. Seeds were examined daily and the emergence of the radicle was considered a parameter for seed germination.

### 4.2. Dose Optimization and Mutagen Treatment

#### 4.2.1. SA and EMS Treatment

Before treatment, seeds were surface-sterilized (as described above) and soaked overnight in sterilized distilled water. After overnight soaking, seeds were subjected to different doses of SA and EMS mutagens independently (0.10 mM to 1.00 mM concentration prepared in 0.1 M phosphate buffer). Mutagen dose optimization was performed by giving different doses of SA and EMS to overnight-soaked seeds in three 50 mL flasks (200 seeds per flask) with mild shaking (100 rpm) at 28 °C. The time duration of the experiment was from 1.5 h to 4 h, followed by 3 washes with distilled water. After washing, seeds were immediately sown in earthen pots. Seed germination was recorded (with 12 days of observation) and only treatments allowing seed germination before 12 days were used for further studies. Overnight-soaked and nontreated seeds were grown and used as a control.

#### 4.2.2. X-ray Treatment

Before the treatment, healthy seeds were surface-sterilized and soaked overnight in sterilized distilled water. Soaked seeds were allowed to germinate in 90 mm sterile glass Petri-plates (Whatman No.1 filter paper moistened with sterile distilled water) to ensure adequate moisture for the seed germination in a seed germinator at 34 ± 1 °C. After the germination of seeds at 7 days, seedlings were subjected to different doses of X-rays, viz., 55 kV, 102 kV, and 125 kV (with 48 mAs, 96 mAs, 128 mAs X-ray dose exposure) electric potential and electromotive force. In the absence of a specific experimental X-ray generator, X-ray treatment was carried out using a SIEMENS MULTIX X-ray system coupled with a Multiphos-450 X-ray generator, which is frequently used in medical diagnostic practice. Here, kV and mAs are the SI units of ‘kilo voltage’ and ‘current exposure time product value’ in terms of X-ray dose exposure. For X-ray mutagenesis, 7-day-old, germinated seedlings in glass Petri-plates (100 seedlings per treatment) were taken and subjected to manual rotating of seedlings for maximum exposure of surface sides to X-ray dose treatment. After the treatment, seedlings were immediately transplanted into pots. Overnight-soaked and nontreated seeds were grown and used as a control.

### 4.3. Plant Growth, Rubisco Assay, and Photosynthetic Activity Estimation

Initially, before conducting mutagenesis treatment, the morphological and physiological growth of the *C. roseus* control plant germinating seedlings were observed for a continuous 12 months, with intervals every 30 days to record plant growth and metabolite kinetics as growth index (GI) in terms of fresh weight, dry weight, and alkaloids content, along with photosynthetic activity in terms of Rubisco activity and chlorophyll content to determine the most productive stage in terms of plant growing and alkaloids.

Later, after mutagenesis and attaining the age of 2 months, growth kinetics of control and mutated plants were monitored in terms of their GI over time using the formula described by Sharma et al. [11] (Supplementary Materials), whereas the Rubisco activity and chlorophyll content of the control and mutated plants were measured. Rubisco activity was measured as described [50] using the spectrophotometric method [51,52]. For chlorophyll estimation, one-gram matured leaves (from each plant sample) were collected and ground in a mortar and pestle by adding 20 mL of 80% (*v*/*v*) acetone as described [53], with some modification. The ground mixture was centrifuged at 5000 rpm for 10 min and the supernatant was collected for chlorophyll estimation. The absorbance of the solutions was measured at 645 nm and 663 nm using a spectrophotometer (Labtronic LT-39, Punchkula, India). Eighty percent (*v*/*v*) of acetone solution was used as blank [53,54]. The chlorophyll a, b, and a + b (total chlorophyll) contents were calculated using the formula as described [55] (Supplementary Materials).

### 4.4. Histological Analysis

Histological analysis was carried out to visualize anatomical differences in leaf epidermal morphology, stomatal count, and vascular system in the leaf, shoot, and root of control and mutated plants. For this, hand-cut transverse sections of the mutated and control plant were prepared as described [27] for microscopic examination. For the stomata counting, the leaf (adaxial) surface was cleaned with wet cotton followed by peeling off the thin epidermal layer and mounting in glycerine on a glass slide with a cover slip. The microscopic observations and photography were carried out using an OMAX microscope fitted with a camera (M837SL-C50U3, San Diego, CA, USA).

### 4.5. Alkaloid Extraction and HPLC Quantification

For HPLC quantification, total alkaloids were extracted from 100 mg oven dried (50–60 °C) leaf samples as described in the protocol (27). The extracted total alkaloid extracts were analyzed through high-performance liquid chromatography (HPLC) for the determination of monomeric alkaloids (vindoline and catharanthine) and dimeric alkaloids (vinblastine and vincristine). The reference standards of vindoline, catharanthine, vinblastine, and vincristine for HPLC were purchased from Sigma-Aldrich, Colombia, SC, USA. Further, Shimadzu Prominencence-I, LC—2030 plus gradient and auto-inject HPLC system with Shimadzu RP-18e reverse-phase HPLC column (Tokyo, Japan) were used for HPLC analysis. For the mobile phase, acetonitrile: ammonium acetate (100 mM, pH 7.3) (50:50 *v*/*v*) solvents (HPLC grade) was used and detection was performed at 254 nm wavelength.

### 4.6. Quantitative Real-Time PCR Analysis of Key MIAs Genes

The quantitative real-time PCR analysis of control and mutated plants was performed. Total RNA was isolated using a Trizol reagent (Genei, India) and treated with Dnase I (Thermo Scientific, Agawam, MA, USA). The qualitative and quantitative estimation of isolated RNA was determined using a NanoDrop spectrophotometer (Thermo Scientific, Agawam, MA, USA). The first-strand complementary DNA (cDNA) was synthesized using the random primers, using the iScript Reverse Transcriptase^TM^ Kit (Bio-Rad, CA, USA). The qRT-PCR program was performed as follows: initial hold at 50 °C for 2 min and 10 min incubation at 95 °C, followed by 40 cycles of 95 °C for 15 s and 55 °C for 1 min. The relative transcript expression of eight candidate MIA pathway genes, viz., *SLS*, *TDC*, *STR*, *SGD*, *T16H*, *DAT*, *PRX1*, and two transcription factors, *ORCA2*, and *WRKY1*, were determined using Livak’s 2^−ΔΔCt^ method [56]. The Ct value (threshold cycle) for each gene was normalized against the Ct from the *C. roseus RSP9* gene (primer list provided in the Supplementary Materials) [57,58].

### 4.7. Statistical Analysis

The experiment was conducted in a completely randomized design and the data presented was expressed as the mean ± standard deviation (SD) of three independent biological replicates. The statistical difference between total alkaloid content, vindoline, catharanthine, and vinblastine were analyzed using applying post hoc Dunnett’s test using SPSS software (IBM, trial version.17.0, Gandhinagar, India).

## 5. Conclusions

Understanding the biosynthesis of key alkaloids and their regulatory mechanisms in the medicinal plant *C. roseus* has seen extensive research in basic and applied sciences. The molecular and biotechnological interventions in *C. roseus* have focussed on large-scale cell-culture methods, genetic engineering approaches, and bioreactor technologies for the enhanced production of MIA alkaloids possessing multi-faceted pharmaceutical attributes; however, physical/chemical mutagenesis in planta and its impact on transcript-metabolome levels has remained a less explored area. In the present study, the sodium azide and ethyl methane sulphonate (chemical mutagens) were used to induce mutagenesis in *C. roseus* and chase its effect over the mutated plants for enhanced MIA (vinblastine) alkaloid production. The anatomical and physio-morphological changes in the mutated plants (compared to the control) and regulation of MIA pathway genes/transcriptional regulation in response to induced chemical mutagenesis were determined. The enhanced accumulation of vinblastine in the mutated plants was found to correlate with the enhanced PRX1 transcript levels, and mutagenesis in *C. roseus* lead to enhanced accumulation of MIA alkaloids (vinblastine, vindoline, catharanthine, and ajmalicine). The present study defines one of the key approaches to enhance in planta content of MIA alkaloids via chemical-induced mutagenesis for pharmaceutical applications.

## Figures and Tables

**Table 1:** Chlorophyll content and Rubisco activity of SA-, EMS-, and X-ray-mutated M_0_ plants.

Mutagen Concentration (mM)	Rubisco Content (Milliunits mL^−1^ mg Protein^−1^) (±SD)	Chlorophyll A (mg/g Fresh Weight) (±SD)	Chlorophyll B (mg/g Fresh Weight) (±SD)	Total Chlorophyll Content (mg/g Fresh Weight) (±SD)
**Control**	**4.40 ± 0.20**	**0.99 ± 0.11**	**0.85 ± 0.08**	**1.84 ± 0.19**
**SA**				
0.1	3.58 ± 0.15	0.95 ± 0.16	0.81 ± 0.13	1.76 ± 0.29
0.2	3.42 ± 0.18	0.81 ± 0.13	0.80 ± 0.12	1.61 ± 0.25
0.3	2.72 ± 0.10	0.74 ± 0.08	0.72 ± 0.08	1.46 ± 0.16
0.4	1.91 ± 0.18	0.70 ± 0.09	0.72 ± 0.09	1.42 ± 0.18
0.5	1.68 ± 0.16	0.60 ± 0.09	0.63 ± 0.07	1.23 ± 0.16
**EMS**				
0.1	3.05 ± 0.13	0.91 ± 0.13	0.82 ± 0.11	1.73 ± 0.24
0.2	2.57 ± 0.16	0.85 ± 0.15	0.80 ± 0.09	1.65 ± 0.24
0.3	2.65 ± 0.13	0.77 ± 0.09	0.76 ± 0.11	1.53 ± 0.20
0.4	1.63 ± 0.13	0.71 ± 0.07	0.70 ± 0.12	1.41 ± 0.19
0.5	1.27 ± 0.12	0.68 ± 0.08	0.64 ± 0.11	1.32 ± 0.19
**X-ray**				
**Control**	2.86 ± 0.35	0.58 ± 0.07	0.51 ± 0.06	1.09 ± 0.13
55 kV (48 mAs)	2.18 ± 0.25	0.45 ± 0.03	0.35 ± 0.04	0.80 ± 0.07
55 kV (96 mAs)	2.82 ± 0.26	0.47 ± 0.03	0.61 ± 0.06	1.08 ± 0.09
55 kV (128 mAs)	2.66 ± 0.24	0.53 ± 0.05	0.38 ± 0.04	0.91 ± 0.09
102 kV (48 mAs)	2.84 ± 0.28	0.51 ± 0.03	0.57 ± 0.04	1.08 ± 0.07
102 kV (96 mAs)	2.28 ± 0.18	0.46 ± 0.06	0.37 ± 0.03	0.83 ± 0.09
102 kV (128 mAs)	1.86 ± 0.16	0.36 ± 0.03	0.28 ± 0.04	0.64 ± 0.07
125 kV (48 mAs)	1.66 ± 0.12	0.34 ± 0.03	0.20 ± 0.03	0.54 ± 0.06
125 kV (96 mAs)	1.56 ± 0.16	0.48 ± 0.06	0.32 ± 0.03	0.80 ± 0.09
125 kV (128 mAs)	1.63 ± 0.14	0.49 ± 0.05	0.33 ± 0.04	0.82 ± 0.09

**Footnote:** SD—Standard deviation, mg—milligram, gm—gram, mL—milliliter, mM—millimolar. No significant difference was observed from the respective control at *p* < 0.05 after applying post hoc Dunnett’s test (*n* = 3).

## Data Availability

Not applicable.

## Supplementary Materials

The following supporting information can be downloaded at: https://www.mdpi.com/article/10.3390/plants11212885/s1: contains Table S1: *C. roseus* plant growth kinetics in relation to alkaloids production; Table S2: *C. roseus* plant chlorophyll content and Rubisco activity in relation to growth kinetics; the formula for chlorophyll estimation and real-time PCR primer list.
