# Supplementary material for: Ethyl Methane Sulfonate and Sodium Azide-Mediated Chemical and X-ray-Mediated Physical Mutagenesis Positively Regulate Peroxidase 1 Gene Activity and Biosynthesis of Antineoplastic Vinblastine in Catharanthus roseus
Source: Plants (Basel). 2022 Oct 28;11(21):2885. doi: 10.3390/plants11212885 (PMC9656251; doi:10.3390/plants11212885)
Supplement: Supplementary file 1 [file plants-11-02885-s001.zip › plants-1954345-supplementary.pdf]

Table S1. *C. roseus* plant growth kinetics in relation to alkaloids production.

Age (Day)	Plant Height (cm)	Fresh Weight (g)	Dry Weight (g)	Alkaloid Content (g)	Vindoline (% dry weight ± SD)	Catharanthine (% dry weight ± SD)	Vinblastine (% dry weight ± SD)
**30**	6±0.56	1.32±0.12	0.09±0.03	0.009±0.001	0.004±0.001	0.008±0.001	ND
**60**	10±0.76	14.12±0.82	1.24±0.21	0.11±0.003	0.012±0.001	0.016±0.002	ND
**90**	14±0.91	24.11±0.96	2.26±0.26	0.28±0.027	0.018±0.001	0.028±0.003	0.001±0.001
**120**	20±1.02	36.32±1.48	3.42±0.43	0.33±0.021	0.021±0.001	0.041±0.003	0.002±0.001
**150**	24±1.21	64.36±2.64	6.68±0.48	0.69±0.023	0.026±0.002	0.056±0.003	0.003±0.001
**180**	28±1.08	96.96±3.28	9.94±0.62	1.03±0.032	0.031±0.003	0.079±0.003	0.004±0.001
**210**	33±1.26	102.01±4.06	10.12±0.68	1.02±0.035	0.030±0.003	0.074±0.005	0.004±0.001
**240**	35±1.21	110.44±4.12	10.26±0.84	0.98±0.024	0.029±0.002	0.066±0.003	0.002±0.001
**270**	36±1.28	108.24±4.66	10.58±1.14	0.96±0.028	0.024±0.002	0.068±0.004	0.002±0.001
**300**	37±1.05	106.36±3.96	9.21±1.01	0.94±0.025	0.022±0.003	0.056±0.004	0.001±0.001
**330**	37±1.0	98.48±3.78	8.46±1.16	0.90±0.034	0.021±0.003	0.042±0.005	ND
**360**	37±1.0	94.38±2.98	8.24±1.12	0.88±0.028	0.019±0.002	0.023±0.005	ND

Table S2. *C. roseus* plant chlorophyll content and Rubisco activity in relation to growth kinetics.

**Age (Day)**	Rubisco Content (milliunits ml^{-1} mg $protein^{-1}$ ± SD)	Chlorophyll A (mg/g fresh weight ± SD)	Chlorophyll B (mg/g fresh weight ± SD)	Total Chlorophyll Content (mg/g fresh weight ± SD)
**30**	0.12±0.01	0.04±0.01	0.03±0.01	0.07±0.02
**60**	0.26±0.03	0.12±0.02	0.08±0.01	0.20±0.03
**90**	1.21±0.03	0.22±0.04	0.18±0.03	0.40±0.07
**120**	1.95±0.19	0.48±0.07	0.36±0.06	0.84±0.013
**150**	2.56±0.24	0.51±0.07	0.49±0.06	1.00±0.10
**180**	2.86±0.35	0.58±0.08	0.51±0.06	1.09±0.13
**210**	2.72±0.32	0.56±0.07	0.45±0.07	1.01±0.15
**240**	2.70±0.24	0.56±0.08	0.46±0.04	1.02±0.12
**270**	2.15±0.19	0.54±0.09	0.41±0.07	0.95±0.16
**300**	1.76±0.21	0.53±0.06	0.41±0.07	0.94±0.13
**330**	1.58±0.24	0.51±0.05	0.36±0.05	0.87±0.10
**360**	1.24±0.23	0.46±0.07	0.38±0.06	0.84±0.13

The formula for calculation chlorophyll a, b, and a + b (total chlorophyll) contents (Arnon 1949).

$$\text{Chlorophyll A} = \frac{12.7\ (\text{A } 663) - 2.69\ (\text{A } 645) \times \text{V}}{1000\ \times W}$$

$$\text{Chlorophyll B} = \frac{22.9\ (\text{A } 645) - 4.68\ (A\ 663) \times V}{1000\ \times \text{W}}$$

$$\text{Totall Chlorophyll} = \frac{20.2\ (\text{A } 663) - 8.02\ (A\ 663)}{1000\ \times \text{W}}$$

Real Time PCR Primer Details

No.	Gene Name	Abbreviation	Primer Direction	Base 5' to 3'direction	NCBI GenBank ID	Amplicon size in bp
1	Secologanin Synthase	SLS	F	CTTCACTCTTGAGAAACTAAAGTCAA	KF415117.1	178
			R	AGTCAATTGTGAGATCCATGAGTT		
2	Tryptophan Decarboxylase	TDC	F	GGTCGAGGATGACGTGGCGGCCGG	MG748691.1	200
			R	ACTCAGACTCAGTGAGTCAACTCGTT		
3	Strictosidine Synthase	STR	F	GCCTTCACCTTCGATTCAACTG	X61932.1	285
			R	GTGGCTAGTTGTGTGGCATACC		
4	Strictosidine B-D-Glucosidase	SGD	F	CATTGGTGAACCGTGCTATG	AF112888.1	186
			R	AGATTGTAGAGTCCAGATGGAACA		
5	Tabersonine 16-Hydroxylase	T16H	F	GCCCAAAACAGCCAATATTCAAACC	FJ647194.1	104
			R	ATGTGATGAGTATGGCCACCGC		
6	Deacetylvindoline Acetyltransferase	DAT	F	CTTCTTCTCATCACGTACCAACTC	AF053307.1	171
			R	ATACCAAACTCAACGGCCTTAG		
7	Peroxidase	PRX1	F	GCGATTCATCAGTGCTGCTGGTGGGA	AM236087.1	278
			R	GTGGAAGGTTTGCTATTGTGTCTGCC		
8	Transcription Factor, Octadecanoid-Responsive *Catharanthus* AP2-domain protein 2	ORCA2	F	TCAACAACGATTTTGATTTTTCA	AJ238740.1	144
			R	TCCGAAGCATAATTTGGTGA		
9	Transcription Factor, WRKYGQK heptapeptide at the N-terminal end	WRKY1	F	GTACTTGGTCCCGACGATATTC	HQ646368.1	146
			R	CGAAACATTCCTTCGTTTGTAAG		
10	Ribosomal Protein 9	RSP9	F	GCGTTTGGATGCTGAGTTGAAG	AJ749993.1	256
			R	GGCGCTCAAGGAAGTTCTCTAC		
